# Genotypes of *Enterocytozoon bieneusi* in Dogs and Cats in Eastern China

**Published:** 2018

**Authors:** Wen-Chao LI, Jie QIN, Kai WANG, You-Fang GU

**Affiliations:** 1. College of Animal Sciences, Anhui Science and Technology University, Fengyang 233100, China; 2. Chuzhou City Vocation College, Fengyang 233100, China

**Keywords:** *Enterocytozoon bieneusi*, Genotype, Dogs, Cats

## Abstract

**Background:**

*Enterocytozoon bieneusi* is a common opportunistic pathogen found in both humans and animals. As companion animals live in close contact with human being, they may act as a zoonotic reservoir and play an important role in transmitting this parasite to humans. We evaluated the prevalence, genotypic diversity and zoonotic potential of *E. bieneusi* in dogs and cats in eastern China during Apr to Dec 2013.

**Methods:**

Fecal specimens from 315 dogs and 143 cats from veterinary hospitals in eastern China were examined in 2015 by internal transcribed spacer (ITS)-based PCR.

**Results:**

*E. bieneusi* was detected in 8.6% of canine and in 1.4% of feline samples. Seven genotypes of *E. bieneusi* were identified, including four known genotypes (PtEb IX, EbpC, Type IV and D) and three novel genotypes, named CHD1, CHD2 and CHD3. The dominant genotype in dogs was PtEbIX (59.3%; n=16/27). Five (CHD1, EbpC, CHD2, D and Type IV) of the seven genotypes were in the so-called zoonotic group 1, whereas genotypes PtEbIX belonged to the dog-specific group and genotypes CHD3 were placed in group 2.

**Conclusion:**

Dogs are predominately infected with host-specific genotypes of *E. bieneusi*, and the finding of several zoonotic genotypes in dogs and cats reminds us of potentially zoonotic transmission of microsporidiosis.

## Introduction

Microsporidia, a phylum including over 1300 species in 160 genera that infect invertebrate and vertebrate hosts, are obligate, intracellular eukaryotes fungal-related parasites (1–3). Among the microsporidian species identified in humans, *Enterocytozoon bieneusi* is typically associated with self-limiting infections in immunocompetent patients and life-threatening chronic diarrhea and wasting diathesis in immunocompromised individuals, such as people with AIDS and organ transplant recipients (4–7). Besides humans, *E. bieneusi* has been also reported in every major animal group (4, 8). Recently, *E. bieneusi* has been detected in drinking water sources and wastewater in America and China, and a foodborne outbreak of microsporidiosis caused by fresh produce contaminated with *E. bieneusi* has been reported in Sweden (9–11). These epidemiological observations have raised public health concerns on the zoonotic nature and water- and food-borne transmission of *E. bieneusi*, however, the reservoir hosts and their precise role in the zoonotic transmission of the parasites are poorly understood (3, 6).

Because of the difficulty in detecting *E. bieneusi* via light microscopy, molecular methods based on sequence and phylogenetic analyses of the internal transcribed spacer (ITS) of the ribosomal RNA (rRNA) gene is regarded as the standard method for species identification and genotyping of *E. bieneusi* isolates (12, 13). To date, over 240 *E. bieneusi* genotypes, clustered into at least nine distinct genetic clusters (groups 1 to 8 and the so-called outlier in dogs), have been defined (3, 6). While a large number of genetically related genotypes identified as group 1 have been isolated from both animals and humans and are generally recognized as having major zoonotic potential, those of the other groups primarily consist of genotypes that are animal host-adapted, as they show a narrow host range and possess little to no zoonotic potential (7).

Molecular epidemiological data from recent studies in China regarding *E. bieneusi* in companion animals report that the organism is commonly found in dogs and cats with considerable genetic polymorphism (13–16). Both host-specific and zoonotic genotypes of *E. bieneusi* were reported in dogs and cats and the majority of the genotypes were found to be potentially zoonotic, belonging to genotype group 1 (13–16). With major socioeconomic development and an improvement in living standards, there has been an increase in the number of dogs and cats being kept as family pets by Chinese families. Eastern China is a relatively affluent rural region and one of the most densely populated areas on the Chinese mainland. There are over three million dogs and cats in Shanghai alone (13) and this large population may increase the risk of infection with parasites carried in the feces of companion animals, including *E. bieneusi*, transmitted to humans. Previous studies have demonstrated a low genetic heterogeneity of *E. bieneusi* in dogs and cats in Shanghai, which is considerably different from the finding of numerous *E. bieneusi* genotypes in similar studies in other parts of China, including Heilongjiang, Sichuan, Chongqing, Shaanxi, Jilin and Henan Provinces (13–16). The facts raised the question of whether the genetic heterogeneity of *E. bieneusi* really is low in dogs and cats in other areas of eastern China and identified a need to further explore this parasite in eastern China.

Therefore, the purpose of the present study was to examine the prevalence of *E. bieneusi* in dogs and cats kept in eastern China, to determine the diversity of circulating *E. bieneusi* genotypes and assess their zoonotic potential.

## Materials and Methods

### Sample collection and processing

From Apr to Dec 2013, 315 canine fecal samples were collected from seven veterinary hospitals in Hefei, Xuanzhou, Chuzhou, Bengbu and Suzhou cities in Anhui Province and Hangzhou in Zhejiang Province. During Mar to Nov 2015, 143 feline fecal samples were collected from four veterinary hospitals in Anhui Province (Lu’an city), Zhejiang Province (Hangzhou city), Jiangsu Province (Yangzhou city) and Shanghai city (Minhang Districts), eastern China, between Mar and Nov 2015. The animals were selected only according to each owner’s willingness to participate in the study and accessibility of animals for sampling. The dogs were divided into two age groups: ≤ 12 months (n = 45) and > 12 months (n = 270). Likewise, 143 cat fecal specimens were divided into two age groups: ≤ 12 months (n = 18) and > 12 months (n = 125), with the remaining cats being of unknown age (n = 39). Overall, 150 dogs were male and 165 were female, while 84 cats were male and 59 were female. Fecal specimens were collected using plastic bags either from the rectum of the animals or from the ground after defecation, submitted to the laboratory and preserved at 4 °C until DNA extraction.

The present study protocol was approved by the Animal Care and Welfare Committee of Anhui Science and Technology University.

### DNA extraction and PCR

Fecal samples were washed three times with distilled water using centrifugation. Genomic DNA was extracted from approximately 200 mg of processed specimen using the Stool DNA Kit (Tiangen, Beijing, China) following the manufacturer’s instructions. The extracted DNA was stored at −20 °C until used in PCR analysis. All the DNA preparations were tested for the presence of *E. bieneusi* using nested PCR amplification of a fragment covering the entire ITS region of the rRNA gene and the primers and the cycling parameters in nested PCR were used (17). In brief, a PCR product of 410 bp was amplified using primers AL4037 (5′-GATGGTCATAGGGATGAAGAGCTT-3′) and AL4039 (5′-AATACAGGATCACTTGGATCCGT-3′) in the primary PCR. A fragment of 392 bp was amplified using AL4038 (5′-AGGGATGAAGAGCTTCGGCTCTG-3′) and AL4040 (5′-AATATCCCTAATACAGGATCACT-3′) for secondary PCR. The reaction conditions for the primary and secondary PCR were identical at the following temperature profiles: initial denaturation at 94 °C for 5 min, followed by denaturation at 94 °C for 30 sec, annealing at 57 °C for 30 sec and extension at 72 °C for 40 sec for 35 cycles, followed by a final extension for 10 min at 72 °C. Two replicates were used in the PCR analysis of each specimen.

### DNA sequence analysis

All positive secondary PCR products were analyzed using agarose gel electrophoresis and visualized with ethidium bromide staining. Products of the expected size were purified with a Biospin Gel Extraction Kit (BIOER, Hangzhou, China) and directly sequenced in both directions on an ABI 377 automated DNA sequencer (Applied Biosystems, Foster City, CA, USA).

All nucleotide sequences obtained in this study were edited with each other and reference sequences downloaded from the Gen-Bank database using the BioEdit v 7.1 (http://www.mbio.ncsu.edu/BioEdit/bioedit.html), aligned using Clustal X 1.83 (http://www.clustal.org/).

Neighbor-joining trees of nucleotide sequences were constructed based on genetic distances calculated by the Tamura 3 parameter model implemented in the program Mega 6.06 (https://www.megasoftware.net/). Representative nucleotide sequences from this study were deposited in GenBank under accession numbers KX869922–KX869926 for *E. bieneusi* isolates in dogs and KX964627– KX964628 for *E. bieneusi* isolates in cats, respectively.

### Statistical analysis

The differences in infection rates in age or sex groups were compared using the χ^2^ test in SPSS version 17.0 (SPSS Inc., Chicago, IL, USA). Differences with *P*<0.05 were considered significant.

## Results

### Occurrence of E. bieneusi in dogs and cats

*E. bieneusi* was detected in 8.6% (27/315) of the 315 canine specimens, including 9.3% (20/215) in Anhui Province and 7.0% (7/100) in Zhejiang Province (Table 1). The differences in infection rates between Anhui Province and Zhejiang Province were not statistically significant (*P*>0.05). Within Anhui Province, Xuancheng (32.0%, 8/25) had a higher prevalence of *E. bieneusi* compared with the other five sampling sites within the province (*P*<0.05). Of the 143 feline fecal specimens, 1.4% (2/143) were positive for *E. bieneusi* and two positive samples were both from Zhejiang Province (Table 1).

**Table 1:** Prevalence and genotype distribution of *Enterocytozoon bieneusi* in dogs and cats in eastern China

***Host and geographic locations***		***No. samples***	***No. positives***	***Positive rate/%***	***genotype(no. of specimens)***
Dog					
Anhui Province	Hefei	29	0	0	
	Xuancheng	25	8	32.0	PtEb IX(6), EbpC(2)
	Fengyang,Chuzhou	17	2	4.5	EbpC(2),CHD1(2)
	Mingguang,Chuzhou	72	2		
	Bengbu	31	6	19.4	PtEb IX(4), CHD2 (2)
	Suzhou	41	2	4.9	PtEb IX(2)
Subtotal		215	20	9.3	PtEb IX(12),EbpC(4),CHD1(2),CHD2 (2)
Zhejiang Province	Hangzhou	100	7	7.0	PtEb IX(4), CHD 3(3)
Total		315	27	8.6	PtEb IX(16),EbpC(4),CHD1(2), CHD2 (2),CHD 3(3)
Cat					
Anhui Province	Liu an	9	0	0	
Zhejiang Province	Hangzhou	81	2	2.5	Type IV (1), D (1)
Jiangsu Province	Yangzhou	39	0	0	
Shanghai city	Minhang	14	0	0	
Total		143	2	1.4	Type IV (1), D (1)

The infection rates based on the age and sex of the dogs and cats are shown in Table 2. There was no significant difference in the infection rates between the two age categories or sex, for both dogs and cats (*P*>0.05).

**Table 2:** Prevalence of *Enterocytozoon bieneusi* in companion dogs and cats, by age and sex, in eastern China

***Host***	***Age(yr)/Gender***	***No. of specimens***	***No. of positives (Positive rate/%)***	***Genotypes (no. of specimens)***
Dog	≤12	45	3(6.7)	PtEb IX(5), CHD1(2)
	>12	270	24(8.9)	PtEb IX(11), EbpC(4), CHD2 (2), CHD 3(3)
	Male	150	12(8.0)	PtEb IX(7), EbpC(3), CHD1(1), CHD2 (2), CHD 3(1)
	Female	165	15(9.1)	PtEb IX(9), EbpC(1), CHD1(1), CHD 3(2)
Cat	≤12	18	0(0)	
	>12	125	2(1.6)	Type IV (1), D (1)
	Male	84	2(2.4)	Type IV (1), D (1)
	Female	59	0(0)	

### E. bieneusi genotypes in dogs and cats

Seven *E. bieneusi* genotypes, comprising four known genotypes (PtEb IX, EbpC, Type IV, D) and three novel genotypes (CHD1, CHD2, CHD3) were identified by ITS sequencing analysis in 29 positive specimens from dogs and cats. In the 27 positive dog specimens, five genotypes were identified, two of known (PtEbIX and EbpC) and three of which were new (CHD1, CHD2 and CHD3). In contrast, the known genotypes (Type IV and D) were found in the two cat specimens that tested positive.

In dogs, the genotype PtEbIX was found in 59.3% (16/27) of samples in both Anhui Province and Zhejiang Province. Additionally, the genotype PtEbIX was detected in 60% of sampling sites in Anhui Province, showing predominance in dogs and a wide geographic distribution.

Genotype EbpC was detected in four dogs and found in two sampling sites in Anhui Province, whereas the remaining three novel genotypes were seen in one specimen each and found only in one sampling site in Anhui Province, respectively. In cats, the two positive specimens belonged to genotype Type IV and D, respectively. The distribution of the genotypes based on the geographic source, age, and sex of the dogs and cats are shown in Tables 1 and 2.

### Genetic relationships

**Fig. 1:**
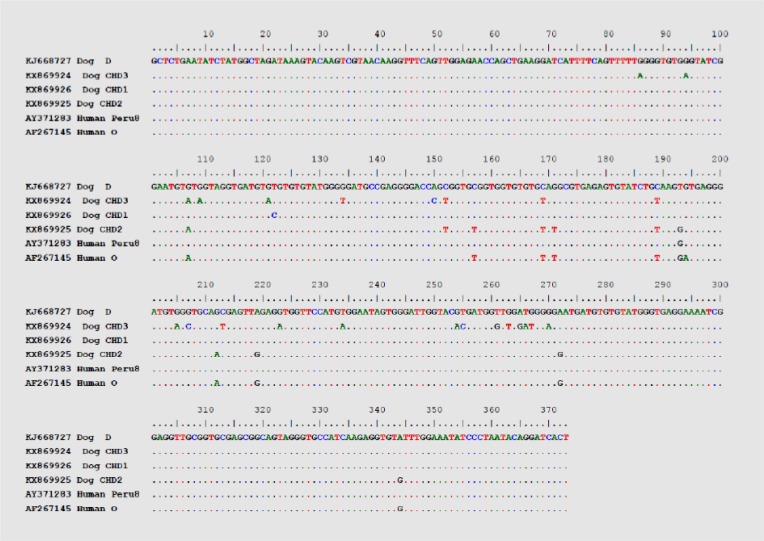
Alignment of ITS sequence of three new genotypes (genotypes CHD1, CHD2 and CHD3) with three genotypes (genotypes D, Peru8 and O) available in the GenBank. Only the nucleotides that differ from those in the reference sequence (KJ668727) are indicated. Dots represent the consensus sequence of all the genotypes

Nucleotide sequence analysis revealed that the novel genotypes CHD1and CHD3 had one single nucleotide polymorphism (SNP) comparable to the two established genotypes D (GenBank accession number KJ668727) and BEB6 (EU153584), respectively. Genotypes CHD1 had two SNPs comparable to genotypes Peru8 (AY371283) and pigEBITS8 (AF348476), respectively. Genotypes CHD2 had two SNPs comparable to genotypes O (AF267145) and H (AF135835), respectively. Similarly, genotypes CHD3 had two SNPs comparable to genotypes BEB7 (EU153585) (Fig. 1).

**Fig. 2:**
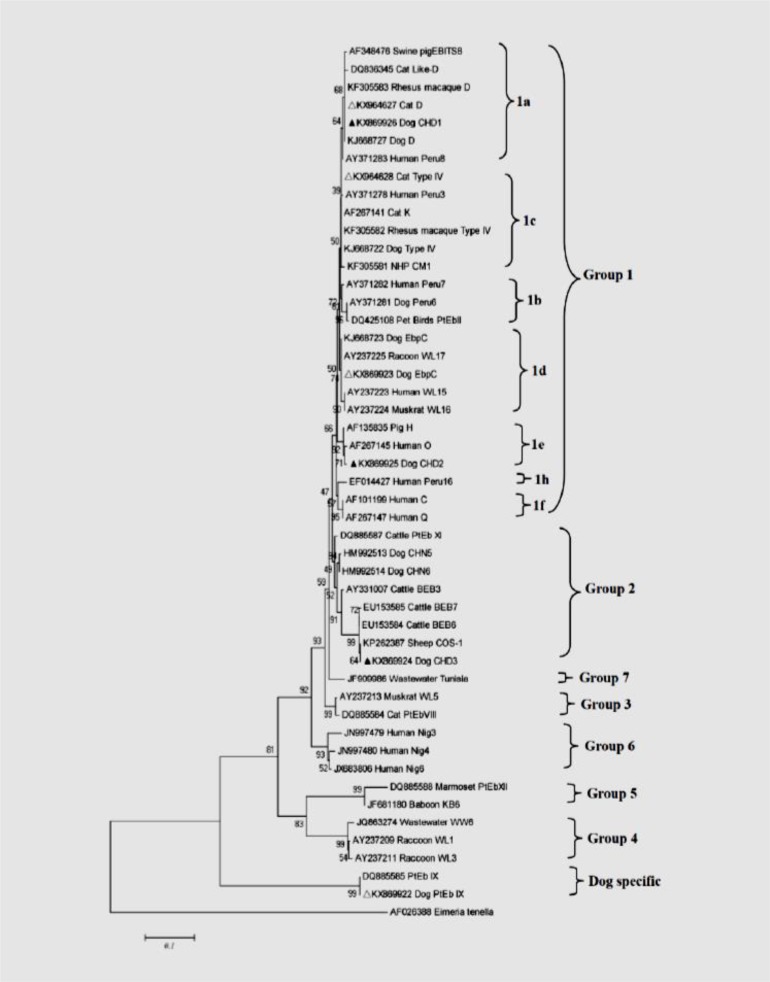
Phylogenetic relationship of *E. bieneusi* genotypes identified in this study and other reported genotypes deposited in GenBank as inferred by a neighbor-joining analysis of the internal transcribed spacer (ITS) sequences based on genetic distances calculated by the Tamura 3 parameter model. The ITS tree was rooted with GenBank sequence AF026388. Each sequence is identified by its accession number, host origin and genotype designation The known and novel genotypes are marked by empty and filled triangles, respectively

Phylogenetic analysis of a neighbor-joining tree (Tamura 3 parameter model) of *E. bieneusi* genotypes showed that five (CHD1, EbpC, CHD2, D, and Type IV) of the seven geno-types belong to the so-called zoonotic group 1 and they were further classed into subgroup 1a, 1d, 1e, 1a and 1c, respectively. However, a large number of dogs (16/27) were infected with host-specific genotype PtEbIX. Interestingly, the new genotype CHD3 was placed in group 2, having previously been designated cattle host specificity (Fig. 2).

## Discussion

*E. bieneusi* was initially recognized as human-specific protozoa, especially in patients with AIDS. However, it has a low host-specificity and many domestic, wild and companion animals may act as a reservoir host. Despite the advances in exploring the genotypic and phylogenetic characteristics in various hosts, including dogs and cats, the reservoir hosts and their precise role in zoonotic transmission remained poorly understood (4–6, 18). In this study, *E. bieneusi* was detected in 8.6% of dogs’ fecal specimens analyzed in eastern China. The results were consistent with previous research that reported the infection rates of *E. bieneusi* ranged from 4.5% to 15.5% in dogs in other parts of China, and Colombia (13–16,19). The prevalence of *E. bieneusi* in cats sampled in the present study (1.4%) was lower than previously reported rates, which ranged from 5.0% to 17.4% in other parts of China, Thailand and Colombia (5, 13, 15–16, 20).

In the present study, there was no significant age-associated difference in *E. bieneusi* infection rates in dogs and cats. This was consistent with observations in Colombia and two surveys in Shanghai city and Henan Province, China (13, 15, 19). There was also no significant correlation between infection rates and sex in dogs or cats, which contradicts a previous report in dogs (19). Infection rate for *E. bieneusi* is often associated with many risk factors, including immune competency of hosts, infection intensity, laboratory diagnosis of infections, sample size, animal management practices, as well as climate and geography, therefore additional efforts should be made to clarify the key risk factors for *E. bieneusi* infection (3).

In the present study, seven genotypes were identified including four known and three novel genotypes. This research determined a moderate degree of genetic diversity in *E. bieneusi* from dogs and cats in eastern China. However, previous studies demonstrated a high degree of genetic diversity in other parts of China, including Heilongjiang, Sichuan, Chongqing, Shaanxi, Jilin and Henan Provinces (14–16). Compared with the present study, a low genetic heterogeneity of *E. bieneusi* was observed in dogs and cats in Shanghai city and only genotypes PtEb IX and D in dogs and genotypes Type IV and D in cats were identified (13).

Four known genotypes (PtEb IX, EbpC, Type IV and D) were previously found in dogs and cats in Thailand, Colombia, Japan, Germany, Portugal, and Shanghai, and Henan, Sichuan and Heilongjiang Provinces in China (5,13,15–16,19, 21–23). The dominant genotype in dogs was PtEbIX, also reported in dogs worldwide and is considered the most common dog-adapted *E. bieneusi* genotype (4,19,21,24). The other three known genotypes, EbpC, Type IV and D, are commonly described in humans and have a wide host range and geographical distribution (4,18). Beyond that, genotypes D and EbpC have also been detected in lake water, wastewater and drinking water sources (9–10, 25). These studies establish the zoonotic nature and public health significance of the three genotypes. Thus, dogs and cats potentially have a role in the zoonotic transmission of *E. bieneusi* genotypes. Furthermore, the genotypes EbpC, Type IV and D have been found in humans, nonhuman primates, dogs, cats and some livestock in China (6, 16, 24, 26, 27). Cross-species transmission of these *E. bieneusi* genotypes maybe occur commonly in China and measures must be taken to prevent the occurrence of cross-transmission and re-infection of *E. bieneusi* between different individuals including human and a variety of animal species (3, 15).

Phylogenetic analysis demonstrated that two of the three new *E. bieneusi* genotypes in this study (CHD1 and CHD2) belong to the so-called zoonotic group 1 and therefore have zoonotic potential and public health importance (2). Of these genotypes, genotype CHD1 is closely related to genotype D with one SNP and CHD2 resembles genotype O and H with two nucleotide differences. This places them in subgroup 1a and subgroup 1e, respectively. Genotype CHD3 has two nucleotide substitutions compared with genotype BEB7, identified in cattle in the United States of America and is placed in the cattle-specific group 2. These results are consistent with previous observations and further support the assertion that the genotypes of group 2 are not cattle specific (14–15, 28, 29).

## Conclusion

Dogs are predominately infected with dog host-specific genotypes and to a certain extent, potentially zoonotic genotypes of *E. bieneusi*. In contrast, cats appear to be infected with predominantly zoonotic genotypes. Consequently, dogs and cats can serve as potential reservoir hosts for zoonotic *E. bieneusi* genotypes and may constitute a risk for public health. The present study highlights the need for future molecular epidemiological investigations in companion animals (dogs and cats) and humans residing in the same location, to elucidate the role of companion animals in the epidemiology of microsporidiosis.
